# RND multidrug efflux pumps: what are they good for?

**DOI:** 10.3389/fmicb.2013.00007

**Published:** 2013-02-05

**Authors:** Carolina Alvarez-Ortega, Jorge Olivares, José L. Martínez

**Affiliations:** Departamento de Biotecnología Microbiana, Centro Nacional de Biotecnología, Consejo Superior de Investigaciones CientíficasMadrid, Spain

**Keywords:** multidrug efflux pumps, host/bacteria interactions, plant/bacteria interactions, quorum sensing, antibiotic resistance, bacterial homeostasis, bacterial virulence

## Abstract

Multidrug efflux pumps are chromosomally encoded genetic elements capable of mediating resistance to toxic compounds in several life forms. In bacteria, these elements are involved in intrinsic and acquired resistance to antibiotics. Unlike other well-known horizontally acquired antibiotic resistance determinants, genes encoding for multidrug efflux pumps belong to the core of bacterial genomes and thus have evolved over millions of years. The selective pressure stemming from the use of antibiotics to treat bacterial infections is relatively recent in evolutionary terms. Therefore, it is unlikely that these elements have evolved in response to antibiotics. In the last years, several studies have identified numerous functions for efflux pumps that go beyond antibiotic extrusion. In this review we present some examples of these functions that range from bacterial interactions with plant or animal hosts, to the detoxification of metabolic intermediates or the maintenance of cellular homeostasis.

## INTRODUCTION

Multidrug resistance (MDR) efflux pumps are relevant elements that contribute to both intrinsic and acquired resistance to toxic compounds in diverse life forms, including humans where they have a role in resistance to anti-cancer drugs (Wu et al., 2011), to bacteria, where they are involved in resistance to antibiotics (Webber and Piddock, 2003; Li and Nikaido, 2004, 2009; Poole, 2005, 2007). Unlike well-known horizontally acquired antibiotic resistance determinants, MDR efflux pumps are usually chromosomally encoded and the structural components of different systems are highly conserved in all members of a given bacterial species (Saier et al., 1998; Paulsen et al., 2001; Saier and Paulsen, 2001; Paulsen, 2003; Baquero, 2004; Andam et al., 2011). MDR systems are ancient elements, present in bacterial genomes long before the use of antibiotics for the treatment of human infections (Martinez et al., 2009a). This, along with their ubiquity in different organisms, suggests that the main function of these elements goes beyond providing resistance to antibiotics. The fact that quinolones, a family of synthetic antibiotics, constitute a common substrate of MDR efflux pumps supports this notion (Alonso et al., 1999; Hernandez et al., 2011b). These observations also suggest that the recent selective pressure imposed by the use of antibiotics is not the main evolutionary driver for MDR efflux pumps (Alonso et al., 2001; Martinez et al., 2009b).

Bacterial MDR efflux pumps can be grouped into five different structural families: the adenosine triphosphate (ATP)-binding cassette (ABC) superfamily (Lubelski et al., 2007), the multidrug and toxic compound extrusion (MATE) family (Kuroda and Tsuchiya, 2009), the major facilitator superfamily (MFS) (Pao et al., 1998), the small multidrug resistance (SMR) family (Chung and Saier, 2001), and the resistance/nodulation/division (RND) superfamily (Murakami et al., 2006; Nikaido and Takatsuka, 2009; Nikaido, 2011). The activity of an efflux pump depends on the different types of energy source each system uses: ABC transporters are fueled by ATP hydrolysis; MFS, RND, and SMR use the proton-motive force and MATE transporters consist of Na^+^/H^+^ drug antiport systems (Piddock, 2006a).

The RND family includes several members that are relevant to antibiotic resistance in Gram-negative bacteria, whereas the MATE family has been mainly associated to resistance in Gram-positive microorganisms (Piddock, 2006a; Vila and Martinez, 2008). This review will focus exclusively on RND efflux systems.

**FIGURE 1:**
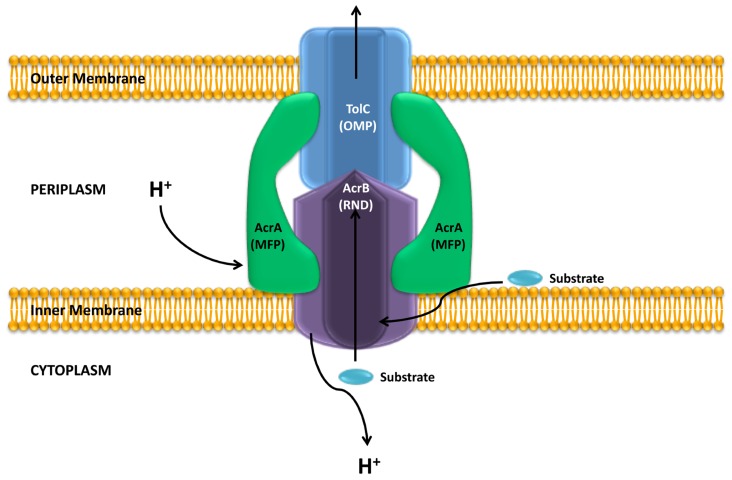
**Structure of an RND efflux pump**. The figure shows a scheme of the structure of the *E. coli* AcrAB-TolC system. As shown, the system is a tripartite complex formed by the inner membrane AcrB protein, the outer membrane protein TolC and the membrane fusion protein AcrA. The activity of the AcrB RND protein is coupled to the proton gradient. It has been shown that these efflux pumps can extrude different compounds form the bacterial cytoplasm and the periplasm. Adapted from Blair and Piddock (2009).

**FIGURE 2:**
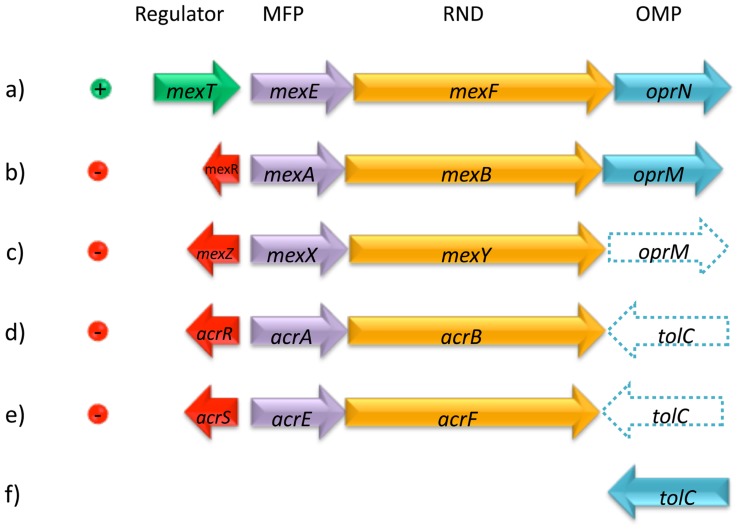
**Representative examples of transcriptional regulation and genetic organization of RND efflux systems**. Local regulators can be either transcriptional activators, such as MexT (a) or transcriptional repressors, such as MexR, MexZ, AcrR, or AcrS (b, c, d, and e). The three structural components may be organized in a single operon, such as in the MexEF-OprN (a) or MexAB-OprM (b) systems; alternatively, a given system may use the OMP from another system, such as in the MexXY system using OprM (c). The OMP component may be located elsewhere in the chromosome, such as TolC (f) and can be used by one or more different systems as in the case of AcrAB (d) and AcrEF (e). MexEF-OprN, MexAB-OprM, and MexXY belong to *P. aeruginosa*; AcrAB and AcrEF belong to *E. coli*.

The crystallographic analysis of AcrB, a model member of the RND family, revealed that this protein forms a homotrimer (Murakami et al., 2002; Nakashima et al., 2011), that associates into a tripartite complex along with an outer membrane protein (OMP, TolC) and a periplasmic membrane-fusion protein (MFP, AcrA; **Figure 1**). Usually the genes encoding for these proteins are found in a single operon, however, the gene encoding for the OMP can also be found elsewhere in the chromosome, as it happens with TolC in *Escherichia coli* (Koronakis et al., 2000, 2004); or is part of an operon encoding for a different efflux pump (**Figure 2**). The *Pseudomonas aeruginosa* RND efflux pump MexXY is an example of the latter, where the system uses the OprM porin encoded in the *mexAB-OprM* operon (**Figure 2**; Mine et al., 1999).

In this review we will address the different functional roles that RND efflux pumps may have in addition to mediating antibiotic resistance. Exhaustive information on structure, regulatory aspects, and antibiotic resistance can be found elsewhere (Saier et al., 1998; Paulsen et al., 2001; Saier and Paulsen, 2001; Paulsen, 2003; Webber and Piddock, 2003; Li and Nikaido, 2004, 2009; Poole, 2004, 2007; Piddock, 2006a; Blair and Piddock, 2009; Nikaido, 2009, 2011; Nikaido and Takatsuka, 2009).

Some of the most relevant roles so far identified include involvement in bacterial virulence (Piddock, 2006b), plant–bacteria interactions (Maggiorani Valecillos et al., 2006), trafficking of quorum sensing molecules (Evans et al., 1998; Kohler et al., 2001), and detoxification processes from metabolic intermediates, and toxic compounds such as heavy metals, solvents, or antimicrobials produced by other microorganisms (Aendekerk et al., 2002, 2005; Ramos et al., 2002; Nies, 2003; Sekiya et al., 2003; Burse et al., 2004a). A comprehensive review of all potential functions identified to date for all RND efflux pumps is beyond the scope of this review. Instead, we would like to discuss some selected examples of the ecological role that these systems may have in the absence of antibiotics. As stated above, we believe that the evolution of bacterial RND efflux pumps has been primarily driven by their physiological functions and not by the selective pressure imposed by the relatively recent human use of antibiotics. We consider the important role RND efflux pumps currently play in antibiotic resistance to be an evolutionary novelty stemming from the aforementioned use of antibiotics by humankind (Martinez, 2008; Baquero et al., 2009).

## REGULATION OF RND EFFLUX SYSTEMS BY NATURAL EFFECTORS

The regulation of bacterial RND efflux systems is often mediated by global and local regulators, resulting in a multilayered control to optimize gene expression in response to specific cues. A number of positive and negative regulators along with their known mechanism of action have been reviewed elsewhere (Grkovic et al., 2002; Li and Nikaido, 2009).

In most cases a transcriptional regulator (typically a repressor) is encoded upstream the operon coding for the efflux pump (**Figure 2**). This local regulator usually keeps expression of the efflux pump at a very low-level. High-level expression can be achieved either through an effector-mediated release of the repressed state or through mutations in one or more regulators (Hernandez et al., 2009, 2011a). Activation may occur at different levels: (1) By inactivation of the local repressor that blocks the expression of the pump’s structural genes such as AcrR in *E. coli* (Ma et al., 1996), MexR in *P. aeruginosa* (Poole et al., 1996; Sanchez et al., 2002c), or SmeT in *Stenotrophomonas maltophilia* (Sanchez et al., 2002a); (2) By activation of a global transcriptional regulator like SoxS, RobA, or RamA in *E. coli* (Martin et al., 2008; Zhang et al., 2008; Perez et al., 2012); (3) By switching on–off one or more steps that interlink regulatory cascades such as MtrR of *Neisseria gonorrhoeae* (Johnson et al., 2011); and (4) Through the emergence and selection of mutations in key genes like *mexT* in *P. aeruginosa* (Kohler et al., 1999).

Multidrug efflux pumps extrude a wide range of substrates. However, the number of effectors regulating them is lower in comparison. Understanding the mechanisms of regulation may help in deciphering the function of RND efflux pumps, since it is expected that different effectors trigger expression only when a given pump is required. RND efflux systems whose expression is controlled by natural inducers normally encountered during the course of infective processes have been studied in detail. Induction of expression by bile salts and fatty acids in enteric bacteria are perhaps the best studied examples of substances capable of modulating expression of these systems.

Expression of the *acrAB* system in *E. coli* is induced by decanoate and unconjugated bile salts usually encountered by the organism in the intestinal tract (Ma et al., 1995). The mechanism involves binding of these effectors to the Rob transcriptional regulator (Rosenberg et al., 2003). Bile salts also induce expression of *acrAB* in *Salmonella*, however, in this case the effector binds the RamA transcriptional regulator (Nikaido et al., 2008). Interestingly, in both cases the inductor is also a substrate for the efflux system, thus allowing the cell to respond quickly to deleterious environmental substances. Additional examples of bile salts-mediated induction include the *cmeABC* system in *Campylobacter jejuni*, the *vexD* gene in *Vibrio cholerae* and various RND-type efflux system genes in *Bacteroides fragilis* (Lin et al., 2005b; Pumbwe et al., 2007). These examples strongly suggest that these systems are relevant to bacterial adaptation for surviving in the gut and that this may be their original function.

In this regard, it has been suggested that some efflux pumps from human commensals and pathogens have evolved to overcome the innate immunity of the host (Blair and Piddock, 2009). For instance, the susceptibility to vertebrate antibacterial peptides in *N. gonorrhoeae* depends on the activity of the MtrCDE RND efflux pump (Shafer et al., 1998). Notably, this efflux pump is required to achieve mutation-driven resistance to penicillin in *N. gonorrhoeae* (Veal et al., 2002) and overexpression mutants present reduced susceptibility to several antibiotics and show an increase in *in vivo* fitness (Warner et al., 2007). MtrCDE (Jerse et al., 2003), enhances experimental gonococcal genital tract infections in female mice, whereas the FarAB-MtrE efflux pump (Lee and Shafer, 1999) is not needed to colonize this environment. It has been suggested that FarAB-MtrE is important for the resistance of *N. gonorrhoeae* to certain long-chained fatty acids that are present in the rectum (Lee and Shafer, 1999). Altogether these studies indicate that *N. gonorrhoeae* harbors efflux pumps each one responding to different environmental cues that enable adaptation for survival in different ecosystems.

Metal cations are another example of natural compounds capable of inducing expression of RND efflux pumps. Metals are required as cofactors in several bacterial processes. However, they are toxic at high concentrations. Consequently, bacteria harbor systems to maintain the cellular metal homeostasis. In some cases, this regulation implies that the efflux pump is involved in the extrusion of these toxic effectors (Nies, 2003). However, in other cases the situation is more complex and the effector is simply an environmental cue that indicates the type of ecosystem surrounding the organism. The *cusCBA* system in *E. coli* and *mtrCDE* system in *N. gonorrhoeae* constitute two of the most studied examples of metal-induced regulation among pathogenic bacteria*.* The CusCBA system confers tolerance to copper and silver ions (Franke et al., 2001; Grass and Rensing, 2001). Both substrates serve as natural inducers for *cusCBA* expression (Franke et al., 2001; Yamamoto and Ishihama, 2005), suggesting that this RND efflux pump may have been first selected to overcome the toxicity of these metals. As stated above, the MtrCDE system is involved in resistance to host-derived antibacterial peptides (Shafer et al., 1998). It was recently reported that *mtrCDE* expression is indirectly regulated by free levels of iron through the regulation of its major transcriptional repressor, MtrR, by the MpeR transcriptional regulator (Mercante et al., 2012). Under the proposed model, expression of the efflux system would increase under iron-limited conditions, a situation that bacteria can encounter over the course of the infection process (Martinez et al., 1990). The *P. aeruginosa* CzcABC efflux system confers tolerance to zinc, cadmium, and cobalt and constitutes another example of metal-induced expression. The regulation occurs through the metal-inducible CzcRS two-component system that is activated in the presence of the system’s substrates or indirectly in the presence of copper (Caille et al., 2007; Dieppois et al., 2012).

## THE ROLE OF EFFLUX PUMPS IN PLANT–BACTERIA INTERACTIONS

The rhizosphere is a complex ecosystem characterized by a high microbial activity that results in a bacterial population density that can be two orders of magnitude higher than in bulk soil (Matilla et al., 2007). The structure of the rhizosphere’s microbiota is governed by the release of nutrients through plant root exudates and by the ecological relationships of the microorganisms present in this ecosystem. A transcriptomic analysis of *Pseudomonas putida* grown in the rhizosphere of maize revealed that the expression of different efflux pumps is induced in this ecosystem (Matilla et al., 2007), thus suggesting a relevant function for the colonization of this environment. Plant exudates have been identified as good effectors of RND efflux pumps, and it has been shown that these secondary metabolites bind regulators of RND efflux pumps such as TtgR (Alguel et al., 2007), the local repressor of the TtgABC system in *P. putida* (Teran et al., 2003). Some compounds produced by plants have antibacterial effects and it has been described the RND efflux pumps are required from the first steps of bacterial plant colonization (Espinosa-Urgel et al., 2000) to survival in plant tissues (Barabote et al., 2003), possibly due to their involvement in protection against these compounds. This is the case of *Erwinia amylovora*, the cause of fire blight disease in rosaceous plants (Eastgate, 2000). The plantlet toxic metabolites naringenin and phloretin are good inducers of the efflux pump *acrAB* in this bacterial species, and *E. amylovora acrAB* mutants are much less virulent that their wild-type counterpart (Burse et al., 2004b). A similar situation occurs in *Agrobacterium tumefaciens*. Coumestrol, an antimicrobial root-exudated flavonoid, is both a substrate and an inducer of expression of the *ifeABR* efflux system (Palumbo et al., 1998). The fact that this system is needed for effective root colonization indicates that it plays an important role in *A. tumefaciens* resistance to plant-produced antimicrobials.

Comprehensive analyses on *Erwinia chrysanthemi* RND efflux pumps revealed that each system may differentially contribute to host specificity. Mutants defective in each of the pumps were differently affected in their virulence in diverse hosts and the susceptibility to plant-produced antimicrobials was specific for each pump (Maggiorani Valecillos et al., 2006). As discussed in the case of *N. gonorrhoeae,* this suggests that each of the several efflux pumps encoded in the genome of a given bacterial species may have a different function. This adaptation does not rely exclusively on the extrusion of toxic antimicrobial plant exudates. For instance, salicylic acid, an important signaling molecule produced by plants (Loake and Grant, 2007), induces the expression of the *E. chrysanthemi* efflux pumps *acrAB* and *emrAB* (Ravirala et al., 2007). This indicates that RND efflux pumps are relevant elements mediating bacteria/plant interactions at different levels that include the response to toxic compounds, host specificity and interspecies signal trafficking. This functional role is not confined to plant-infective bacteria. Mutants of the mutualistic symbiont *Rhizobium etli* lacking the RmrAB efflux pump form fewer nodules on its host *Phaseolus vulgaris* than the corresponding wild-type strain (Gonzalez-Pasayo and Martinez-Romero, 2000). Similarly, the SmeAB efflux pump plays an important role in the nodulation competitiveness in *Sinorhizobium meliloti* (Eda et al., 2011). The effect of efflux pumps on plant–bacteria interactions can be host-specific. For instance, BdeAB from *Bradyrhizobium japonicum* is needed for the symbiotic nitrogen-fixation activity on soybean, but not on other host plants such as mung bean and cowpea (Lindemann et al., 2010).

## THE ROLE OF EFFLUX PUMPS IN BACTERIAL VIRULENCE

From a clinical point of view, antibiotic resistance could be considered as a colonization factor since only those organisms surviving within a treated patient will be able to cause an infection (Martinez and Baquero, 2002). However, in this section we would like to address the direct role that RND efflux pumps play in the virulence of different human pathogens. As mentioned in a previous section, the expression of different RND efflux pumps is triggered by human-produced compounds, and they contribute to the colonization of different environments in the human host. Although the role of efflux pumps on virulence has been studied for several organism (Piddock, 2006b), only in a few cases comprehensive studies including different systems from a single bacterial species have been performed. Below we discuss some of these examples

## *Vibrio cholerae* RND EFFLUX PUMPS AND VIRULENCE

*Vibrio cholerae* possesses six different operons encoding for RND-type efflux systems: *vexAB*, *vexCD* (*breAB*), *vexEF*, *vexGH*, *vexIJK*, and *vexLM* (Kitaoka et al., 2011). While different RND efflux systems often share an OMP, it is rather common that operons encode for a cognate OMP for each system (**Figure 2**). In the case of *V. cholerae,* it seems that all six different RND efflux systems operate with the same OMP, encoded by the *tolC* gene (Bina et al., 2008; Cerda-Maira et al., 2008).

During the course of *V. cholerae* infections, bacteria colonize primarily the small intestine, where they penetrate the mucus lining coating the intestinal epithelium. In addition to factors produced by the innate immune system, the intestinal environment is rich in substances such as bile salts and organic acids that are capable of inhibiting bacterial growth (Reidl and Klose, 2002). Predictably, four *V. cholerae* RND efflux systems have been implicated in *in vitro* resistance to bile salts and detergents similar to detergent-like molecules the organism is likely to encounter during colonization of the intestinal epithelium.

Susceptibility studies with single and multiple mutant combinations revealed that VexB has broad substrate specificity and that it is the primary RND efflux system responsible for resistance to bile salts *in vitro* (Bina et al., 2008). VexD, VexK, and VexH have also been implied in resistance to bile salts, which denotes redundancy among the different RND efflux systems (Taylor et al., 2012). Moreover, the expression of *vexD* is induced in the presence of bile salts (Cerda-Maira et al., 2008). The VexK and VexH contribution to bile salts resistance is only evident in a Δ*vexBD* double mutant background, which suggests a supportive role for VexK and VexH. However, as Taylor et al. (2012) point out, this hierarchy might be limited to their *in vitro* experimental conditions. In fact, the increasing attenuation levels displayed by combination mutants in *in vivo* colonization experiments (Δ*vexBDK* Δ*vexBDH* Δ*vexBDHK* < ΔRND), suggest that VexH plays a more relevant role than VexK during the infection process.

RND efflux systems are also required for optimal expression of the genes encoding for two of the most important *V. cholerae* virulence factors: cholera toxin (CT) and the toxin-coregulated pilus (TCP). A ΔRND mutant exhibited decreased transcription of the *tcpA* and *toxT* genes, the latter encoding for a transcriptional activator responsible for transcription of the genes encoding for CT, and a concomitant decrease in CT and TCP production (Bina et al., 2008). While VexB is able to complement this phenotype, a *vexBDHK* still exhibits a decrease in CT and TCP, thus suggesting a role for VexM and VexF in virulence factor production (Bina et al., 2008; Taylor et al., 2012).

The mechanism through which the *V. cholerae* RND efflux systems modulate the production of virulence factors has not been elucidated. However, it has been proposed that deletion of systems with redundant functions could lead to the accumulation of a low molecular weight molecule that normally functions as a negative effector molecule involved in fine-tuning the expression of the affected virulence factors (Taylor et al., 2012). *V. cholerae* inhabits aquatic environments where it normally grows associated with zooplankton or egg masses (Reidl and Klose, 2002). It is possible that some of the RND efflux systems have dedicated functions specific to this portion of the organism’s life cycle. This may be particularly true for VexM and VexF, for which no function in resistance to bile salts and antimicrobials has been identified to date.

## *Mycobacterium tuberculosis* RND EFFLUX PUMPS AND VIRULENCE

The *M. tuberculosis* genome possesses 13 different genes encoding for RND proteins (Cole et al., 1998). Several domains in these proteins are unique to mycobacteria and are thus designated as MmpL (Mycobacterial membrane protein Large). Four *mmpL* genes appear to be in operons also containing an *mmpS* gene. The latter are predicted to encode for proteins equivalent to the MFPs in other bacterial RND systems (Domenech et al., 2005).

In spite of the documented *M. tuberculosis* resistance against first and second line antimicrobial therapy, none of the RND systems have been associated with antibiotic efflux to date, the only possible exception being MmpL7, which is capable of conferring isoniazid resistance when overexpressed in *Mycobacterium smegmatis* (Pasca et al., 2005; De Rossi et al., 2006; da Silva et al., 2011)*.* Moreover, deletion mutants created in 11 *mmpL* genes failed to exhibit significantly altered drug susceptibility in *M. tuberculosis* (Domenech et al., 2005).

The primary role of most MmpL proteins appears to be the transport of lipids to be incorporated on the cell envelope. The complex mycobacterial cell wall is composed of peptidoglycan, arabinogalactan, and mycolic acids, the surface of which is covered by non-covalently associated lipids that include trehalose monomycolate (TMM), trehalose dimycolate (TDM), sulfolipids, phenolic glycolipids, and phthiocerol dimycocerosates (PDIMs; Tahlan et al., 2012). These lipids play important roles in protection against host-derived toxic molecules, bear an immunomodulatory activity and contribute to *M. tuberculosis* pathogenicity (Neyrolles and Guilhot, 2011). Lipid transport functions have been ascribed to MmpL3, MmpL7, and MmpL8, and in some cases deletion mutants have demonstrated the contribution of additional MmpL proteins to host survival and pathogenicity.

The inability to create an *mmpL3* deletion mutant combined with its absence in transposon mutant collections suggests that this gene is essential to *M. tuberculosis* (Domenech et al., 2005; Lamichhane et al., 2005). A recent study aimed at identifying the target of a novel *M. tuberculosis* antibiotic found data that suggests that MmpL3 transports TMM out of the cell and that its inhibition prevents the incorporation of *de novo*-synthesized mycolic acids into the cell envelope (Tahlan et al., 2012).

MmpL7 is required for PDIM transport to the cell surface and was the first MmpL protein implicated in lipid transport in *M. tuberculosis* (Cox et al., 1999). In addition, MmpL7 appears to function as a scaffold for the PpsE polyketide synthase required for the final step of phthiocerol synthesis, thus coupling transport and synthesis (Jain and Cox, 2005). At least two different studies have determined that *mmpL7* mutants display an attenuation phenotype in murine virulence models (Cox et al., 1999; Domenech et al., 2005). MmpL8 has been implicated in the transport of the SL-N, a precursor of the SL-1 sulfolipid, with a similar mechanism to that of MmpL7 where synthesis and transport appear to be coupled (Converse et al., 2003; Domenech et al., 2004). *mmpL8* mutants also display attenuated lethality in murine virulence models (Converse et al., 2003; Domenech et al., 2004, 2005).

Domenech et al. (2005) determined that an *mmpL4* mutant has both impaired growth kinetics and impaired lethality in a virulence murine model. The same study determined that while an *mmpL11* mutant shows a growth pattern similar to that of the wild-type during the active growth phase, the mutant is attenuated during the course of chronic infections in an *in vivo* model. No substrate has been identified for these transporters. A role in heme uptake has been recently proposed for MmpL11 and such a function would be in line with the attenuated virulence phenotype observed with an *mmpL11* mutant (Tullius et al., 2011). Furthermore, a role in extrusion of host-derived antimicrobials similar to that observed for *V. cholerae* RND efflux systems cannot be ruled out for those MmpL proteins that appear to be involved in the *M. tuberculosis* infection process.

## *Helicobacter pylori* RND EFFLUX PUMPS AND VIRULENCE

The gastric colonizer *Helicobacter pylori* possesses three different operons encoding for RND efflux systems (Tomb et al., 1997). Over the years the systems have received different nomenclatures that may often lead to confusion when revising the literature: hp0605–hp0607 is also referred to as *hefABC*; hp0969–hp0971 was originally denominated as *hefFDE* and is currently known as *cznABC*; finally, the system encoded by hp1329–hp1327 was originally named *hefIHG* and currently hp1329 and hp1328 are known as *czcA* and *czcB*, respectively, while hp1327 is known as *crdB.*

Bina et al. (2000) initially assessed *in vitro* and *in vivo* expression profiles of each system as well as the individual contribution to intrinsic antibiotic susceptibility. The study revealed that hp0607 (*hefC)* and hp0969 (*hefF)* are expressed both in *in vivo* and *in vitro,* while hp1329 (*hefI*) is only expressed* in vivo.* Knockouts in each system failed to identify a contribution to intrinsic antibiotic susceptibility with 19 different compounds. However, overexpression of selected components has been associated with antibiotic resistance and different studies revisiting the contribution of each system to antibiotic susceptibility determined that hp0607 (*hefC*) and hp0605 (*hefA*) are involved in intrinsic antibiotic resistance to diverse antibiotics (Kutschke and de Jonge, 2005; Liu et al., 2008; Hirata et al., 2010; Tsugawa et al., 2011).

*H. pylori* is exposed to bile salts resulting from reflux into the human stomach; bile salts have an inhibitory effect on *H. pylori* growth, yet the ability to thrive in the presence of a bile gradient suggests that this organism has bile resistance mechanisms in place (Worku et al., 2004; Shao et al., 2008). HefC was recently found to play a role in resistance to bile salts and ceragenins (synthetic bile salt derivatives with antimicrobial activity; Trainor et al., 2011). A *hefC* mutant exhibited increased susceptibility to deoxycholate, cholate, glycodeoxycholate, taurodeoxycholate, taurocholate and to ceragenin 11(CSA 11); while no changes in susceptibility were observed with mutants in the other two efflux systems. Moreover, HefC appears to have substrate specificity for bile salts, since no change in susceptibility was observed with detergents. Although direct efflux of bile salts through HefC has not been experimentally demonstrated yet, it is likely that this system contributes to *H. pylori* successful colonization of bile-containing environments.

During the course of gastric colonization, *H. pylori* is exposed to additional environmental stresses, including low pH gradients (4.0–6.0) and acid shock. Acidic environments impact the bioavailability of metals like iron and nickel, which play an essential role in bacterial metabolism. In addition, environmental metal fluctuations are expected to arise from damaged epithelium and diffusion from ingested food (Stoof et al., 2008). Maintaining a cytoplasmic metal homeostasis is crucial to bacteria, as excessive concentrations can lead to severe cellular damage. The other two *H. pylori* RND efflux systems are involved in metal efflux.

The system encoded by hp1327–1329 (*crdB, czcB,* and *czcA*) constitutes a novel copper efflux pump. Expression of hp1329 is induced in the presence of copper and growth of hp1327 and hp1328 mutants is inhibited in the presence of this metal (Waidner et al., 2002). The same study found that expression of hp1326 (renamed as *crdA*), encoding for a secreted protein, is strongly induced in the presence of copper and growth of an hp1326 mutant was also impaired in the presence of copper. hp1326 is transcribed as a monocistronic unit, but is believed to constitute a copper resistance system along with hp1327–1329. A follow up study revealed that copper-mediated expression of hp1326 requires the CrdRS two-component system (Waidner et al., 2005); the study did not address expression of hp1327–1229. Mutants lacking the CrdRS system are unable to colonize the stomach of mice (Panthel et al., 2003). This suggests that hp1326 and hp1327–1329 might play an important role during the infective process of *H. pylori.*

The RND efflux system encoded by hp0969–0971 (renamed as *cznABC*) has been implicated in cadmium, zinc, and nickel resistance (Stahler et al., 2006). Stahler et al. (2006)** showed growth inhibition of individual mutants in the presence of these metals. The *H. pylori* urease, a nickel-containing enzyme, is an essential colonization factor that enables survival in acidic conditions. Urease activity and expression is regulated in response to nickel availability (van Vliet et al., 2001), accordingly, *cznC* and *cznA* mutants exhibited enhanced urease activity (Stahler et al., 2006). The authors propose that the *cznABC* system plays an important role in fine-tuning urease activity, as nickel efflux reduces activity, while cadmium and zinc efflux prevents inhibition of this enzyme. High urea concentrations are toxic at neutral pH, therefore, untimely activation of this enzyme resulting from perturbations in metals homeostasis can be detrimental to the cell (Meyer-Rosberg et al., 1996; Rektorschek et al., 1998). The inability of *cznA*, *cznB*, and *cznC* mutants to achieve gastric colonization in a gerbil animal model and the failure of a *cznA* mutant to survive in acidic conditions might be linked to urease activity (Bijlsma et al., 2000; Stahler et al., 2006).

## EFFLUX PUMPS AND GLOBAL BACTERIAL PHYSIOLOGY

One of the putative functions of RND efflux pumps is detoxification from detrimental intermediates derived from bacterial metabolism (Neyfakh, 1997). Studies on this subject have been mainly performed using mutants that overproduce RND efflux pumps. It is conceivable that overexpression of these elements might cause a metabolic burden on bacterial populations (Martinez et al., 2007, 2011; Andersson and Hughes, 2010). Indeed, different publications have shown that overproduction of RND efflux pumps may impact bacterial physiology (Sanchez et al., 2002b; Ruiz-Diez et al., 2003; Alonso et al., 2004; Linares et al., 2005; Lertpiriyapong et al., 2012; Olivares et al., 2012). Moreover, the uncontrolled production of these elements can affect the ability of pathogenic bacteria to infect experimental animal models, seriously impairing their virulence (Cosson et al., 2002; Hirakata et al., 2002; Warner et al., 2007; Lertpiriyapong et al., 2012; Perez et al., 2012).

The energy expenditure required to constantly maintain the activity of an efflux pump could lead to a fitness reduction upon overproduction of these elements. However, our group has recently shown that overproduction of the *P. aeruginosa* MexEF-OprN efflux system does not produce a fitness cost as measured in classical competition tests, although it alters several physiological aspects, including elements relevant for *P. aeruginosa* virulence such as Type III and Type VI secretion (Tian et al., 2009a,b; Olivares et al., 2012). Notably, this effect is specific to each pump and might be associated to their functional role, as overexpression of either MexAB-OprM of MexXY does not produce the same effect (Linares et al., 2005).

As mentioned before, efflux pumps might be involved in the elimination of endogenous toxic compounds. The *P. aeruginosa* MexGHI-OpmD efflux system might be implicated in the extrusion of anthranilate, a toxic intermediate of the *Pseudomonas* quinolone signal (PQS) synthetic pathway (Aendekerk et al., 2002, 2005; Sekiya et al., 2003), whereas MexEF-OprN extrudes kynurenine, another intermediate in the same pathway (Olivares et al., 2012). A recent study has shown that kynurenine and its derivatives have relevant effects in different human diseases, including modulation of the activation of glutamate and nicotinic receptors, the modification of the immune response in situations of inflammation and infection, and the generation and removal of reactive oxygen species (Stone et al., 2012). Any potential impact that the constant extrusion of kynurenine by a MexEF-OprN overexpression mutant may have on the pathogenic behavior of *P. aeruginosa* remains to be established.

*Pseudomonas* quinolone signal is one of the quorum sensing (QS) signals produced by *P. aeruginosa* (Mcknight et al., 2000). Strains overexpressing MDR efflux pumps capable of extruding QS signals or their intermediates are likely to be impaired in the QS response. Indeed, overexpression of MexEF-OprN impairs the QS response of *P. aeruginosa* (Kohler et al., 2001; Olivares et al., 2012). Previous studies also showed that MexAB-OprM likely extrudes the 3O12-HSL QS signal (Evans et al., 1998; Pearson et al., 1999), and that overproduction of this efflux pump reduced the expression of selected QS-regulated genes. The *P. aeruginosa* QS regulon comprises approximately 5% of this organism’s genome (Schuster et al., 2003); including several genes involved in virulence. Expression of some of these genes might be energetically costly. However, once the QS signals reach a specific threshold, expression of the regulon is maintained. It has been suggested that being signal-blind can be a good adaptive strategy to avoid this energetic burden (Haas, 2006). Whether the efflux pump-mediated extrusion of QS signals may be beneficial to *P. aeruginosa* under specific conditions remains to be determined.

Efflux pumps may also compensate for the effects that other bacterial elements may have on the organism. This might be the case of *C. jejuni,* a leading cause of food-borne enterocolitis worldwide (Ruiz-Palacios, 2007). As an intestinal pathogen this bacterium must overcome the antimicrobial effects of the bile salts secreted into the intestinal tract (Hofmann and Eckmann, 2006). The RND-type efflux pump CmeABC confers resistance to a broad range of antibacterial substances including bile salts, fatty acids, and detergents (Lin et al., 2005a). On the other hand, it has been demonstrated that the type VI secretion system (T6SS) plays a key role in the colonization of the intestinal tract (Lertpiriyapong et al., 2012). The activation of the T6SS may enable bile salts to enter inside the bacterium through the open secretion channel (Bidlack and Silverman, 2004); and this can compromise bacterial viability and infective capability. Bile salts trigger the expression of the CmeABC efflux pump; which extrudes the bile salts immediately outside the cell thus alleviating the entrance through the T6SS (Lin et al., 2005b). The functional interaction between the T6SS and CmeABC might be crucial for intestinal colonization by *C. jejuni*, thus playing a key role in the virulence of this bacterial pathogen (Lertpiriyapong et al., 2012).

Given the integration of RND efflux systems in bacterial metabolic networks, it is not surprising that their regulation is also incorporated in global regulatory networks. Global regulators such as MarA, RamA, and SoxS can activate the expression of efflux pumps such as AcrAB-TolC in *E. coli* and in additional *Enterobacteriaceae* (Davin-Regli et al., 2008). Similarly, the pleiotropic regulator MgrA (Luong et al., 2006) controls autolysis, virulence, biofilm formation, and efflux pump activity in *Staphylococcus aureus* (Ingavale et al., 2003, 2005; Truong-Bolduc et al., 2003, 2005; Trotonda et al., 2008). The control of efflux pumps by this global regulator is specific for each pump. Increased expression of *mgrA in vivo* in a subcutaneous abscess model upregulates expression of the *norB* and *tet38* efflux pumps, whereas expression of *norA* and *norC* is downregulated (Ding et al., 2008). The relevance of these pumps for the *in vivo* growth of *S. aureus* has been studied; *norB* and *tet38* defective mutants present a growth defect in a mice abscess model and the phenotype was not attributable to a staphylococcal stress response (Deng et al., 2012).

MexT, the transcriptional activator of MexEF-OprN in *P. aeruginosa* (**Figure 2**), constitutes another example of global regulation. MexT regulates the expression of several *P. aeruginosa* genes (Tian et al., 2009a). A portion of this regulation is mediated by the activity of the pump through the extrusion of a precursor of the PQS QS signal, and the concomitant impairment of the QS response (Olivares et al., 2012). However, the expression of other genes is directly regulated by MexT (Tian et al., 2009a). A recent study demonstrated that MexT functions as a redox-responsive regulator (Fargier et al., 2012), indicating that it might be involved in controlling cellular redox homeostasis. The fact that a local transcriptional regulator of an efflux pump behaves as a global regulator further supports the involvement of these elements in general processes of bacterial physiology and not simply as a response to the presence of antibiotics in the environment.

## CONCLUDING REMARKS

The emergence of antibiotic resistance in bacterial human pathogens is a very recent process in the evolutionary timescale. It is often assumed that resistance genes have been mainly originated in antibiotic producers where they play a detoxification role (Benveniste and Davies, 1973; Webb and Davies, 1993; Davies, 1997). However, in the few cases where the origin of resistance genes has been tracked, the original hosts are not antibiotic producers. The QnrA gene from *Shewanella algae* constitutes a prime example, as it confers resistance to quinolones, which are synthetic antibiotics (Poirel et al., 2005). This indicates that, at least in some cases, antibiotic resistance would be an emergent function that has been recently selected due to the use of antibiotics for treating infections (Martinez, 2008, 2009a,b; Baquero et al., 2009; Fajardo et al., 2009). As we have seen in this review, MDR efflux pumps also fall within this category, since they exhibit multiple functions relevant to bacterial physiology in addition to mediating antibiotic resistance. A complete understanding of these functions is important in order to define the networks that connect antibiotic resistance with other basic physiological processes (Linares et al., 2010; Martinez and Rojo, 2011), both during the course of infections and in natural, non-clinical ecosystems.

## Conflict of Interest Statement

The authors declare that the research was conducted in the absence of any commercial or financial relationships that could be construed as a potential conflict of interest.
